# Transcriptional landscape of psoriasis identifies the involvement of IL36 and IL36RN

**DOI:** 10.1186/s12864-015-1508-2

**Published:** 2015-04-19

**Authors:** Maris Keermann, Sulev Kõks, Ene Reimann, Ele Prans, Kristi Abram, Külli Kingo

**Affiliations:** Department of Dermatology, University of Tartu, Tartu, Estonia; Department of Dermatology, Tartu University Hospital, Tartu, Estonia; Department of Pathophysiology, Centre of Translational Medicine, University of Tartu, 19 Ravila Street, 50411 Tartu, Estonia; Department of Reproductive Biology, Estonian University of Life Sciences, Tartu, Estonia

**Keywords:** Psoriasis, Transcriptome, Gene expression profiling, High-throughput nucleotide sequencing, Functional genomics

## Abstract

**Background:**

In present study we performed whole transcriptome analysis in plaque psoriasis patients and compared lesional skin with non-lesional skin and with the skin from healthy controls. We sequenced total RNA from 12 lesional (LP), 12 non-lesional (NLP) and from 12 normal (C) skin biopsies.

**Results:**

Compared with previous gene expression profiling studies we had three groups under analysis - LP, NLP and C. Using NLP samples allows to see the transcriptome of visually normal skin from psoriasis patient. In LP skin S100A12, S100A7A, LCE3E, DEFB4A, IL19 were found up regulated. In addition to already these well-described genes, we also found several other genes related to psoriasis. Namely, KLK9, OAS2, OAS3, PLA2G, IL36G, IL36RN were found to be significantly and consistently related to the psoriatic lesions and this finding is supported also by previous studies. The genes up-regulated in the LP samples were related to the innate immunity, IL17 and IL10 networks. In NLP samples innate immunity and IL17 network were activated, but activation of IL10 network was not evident. The transcriptional changes characteristic in the NLP samples can be considered as a molecular signature of “dormant psoriasis”.

**Conclusions:**

Taken together, our study described the transcriptome profile characteristic for LP and NLP psoriatic skin. RNA profile of the NLP skin is in between the lesional and healthy skin, with its own specific pattern. We found that both LP and NLP have up-regulated IL17 network, whereas LP skin has up regulated IL10 related cytokines (IL19, IL20, IL24). Moreover, IL36G and IL36RN were identified as strong regulators of skin pathology in both LP and NLP skin samples, with stronger influence in LP samples.

**Electronic supplementary material:**

The online version of this article (doi:10.1186/s12864-015-1508-2) contains supplementary material, which is available to authorized users.

## Background

Psoriasis is one of the most prevalent chronic inflammatory disease affecting skin and joints [1]. The disease affects 2–3% of the population worldwide and it can have variable clinical course and severity [2]. The commonest form of psoriasis is plaque psoriasis and chronic, symmetrical, silvery-scaled plaques characterize this form [1,3]. Although the cause of psoriasis is unknown, it is complex disease with multifactorial pathogenesis where several genes interact and eventually induce active disease [4-6].

Whole-genome analysis of RNA provides useful tool to study complex disorders and to find common pathways related to the phenotype. Studies of the psoriatic transcriptome with microarrays and RNA-seq technologies have revealed very large number of differentially expressed genes (DEGs) in lesional skin [7-12]. With microarrays more than 1000 genes have been found differentially regulated between psoriatic and normal control skin [8,10]. These studies have resulted in the new genes and candidates involved in the pathogenesis of psoriasis. A more recent study has found that 80% of significantly elevated genes in psoriasis lesions are related to the keratinocyte activity and infiltration by T-cells and macrophages [9]. In addition to genechips, recently evolved RNA-seq provides more comprehensive overview about the transcriptional landscape. RNA-seq is able to detect new transcripts and splicing forms that are undetectable with other tools. There have been only two RNA-seq based studies on psoriasis so far [12,13]. Both of these studies described several new transcripts involved in the psoriasis. In one study three pairs of lesional and normal skin samples from psoriatic individuals were analysed [13]. In the most recent study based on biopsies of 92 psoriatic patients and 82 normal individuals 3,577 DEGs between lesional and normal skin were described [12]. In present study, we performed RNA-seq transcriptome analysis of 12 paired lesional (LP) and non-lesional skin (NLP) samples with 12 samples from healthy controls (C). Differential gene expression analysis was combined with functional network annotation. As a result we found gene expression pattern characteristic for the psoriatic lesions and described the molecular signature of the non-lesional skin of psoriatic patient.

## Methods

### Patients and controls

The Ethics Review Committee on Human Research of the University of Tartu approved the protocols and informed consent forms used in this study. All the participants signed a written informed consent. The patients and control subjects in the study were unrelated Caucasians living in Estonia. Unrelated patients with plaque psoriasis from the Dermatology Clinic of Tartu University Hospital were included in the study. Sex- and age-matched (+/− 10 years) control subjects were recruited among patients with melanocytic nevi at the dermatologic outpatient clinic. Healthy volunteers were free from inflammatory dermatoses and without a positive family history of psoriasis. The main characteristics of the psoriasis patients are presented in Table 1.

**Table 1:** **Characteristics of psoriasis patients in present study**

**Patient**	**Age, years**	**Sex**	**AoO (duration), years**	**PASI**	**Nail involvement**	**PsA**	**Scalp involvement**	**Flexural ininvolvement**	**Treatment**
P847	19	M	18 (0.5)	12.5	No	No	Yes	No	No
P652	25	M	13 (12)	18.4	Yes	No	Yes	Yes	No
P844	27	M	18 (9)	8.8	No	No	Yes	No	No
P848	29	M	26 (3)	3.7	No	No	Yes	No	No
P840	49	M	15 (34)	6.0	No	No	Yes	Yes	Calcipotriol + betamethasone
P845	52	M	22 (30)	14.0	Yes	No	Yes	Yes	No
P851	60	M	56 (4)	10.8	No	No	Yes	No	No
P853	28	F	28 (0.5)	23.3	No	No	Yes	No	No
P849	37	F	30 (7)	4.7	No	No	No	No	Topical steroid
P856	54	F	14 (40)	7.3	No	Yes	Yes	Yes	Topical steroid
P843	57	F	53 (4)	12.6	No	No	No	No	Topical steroid
P846	58	F	57 (1)	15.2	No	No	Yes	No	Topical steroid

AoO = Age of Onset, M = male, F = female, PASI = Psoriasis Area and Severity Index, PsA = psoriatic arthritis.

Two 4 mm punch biopsy specimens were taken from patients with psoriasis, one from the lesional (LP sample) and another from distant non-sun-exposed nonlesional (NLP sample) skin. One 4 mm punch biopsy specimen was taken from non-sun-exposed skin of each control subject (C sample). All biopsy specimens were instantly frozen in liquid nitrogen and stored at -80C° until RNA extraction.

### RNA sequencing

Precellys24 tissue homogenizer (Bertin Technologies, France) with Cryolys adaptor was used to homogenise biopsy specimens. Total RNA was extracted with RNeasy Fibrous Tissue Mini Kit (Qiagen, Valencia CA, USA) according to the manufacturer’s protocol. Isolated RNA was dissolved in RNase free water and stored in -80C°. The quality of total RNA was evaluated with Agilent 2100 Bioanalyzer and the RNA 6000 Nano Kit (Agilent Technologies Inc., CA, USA). The average RNA integrity number (RIN) of samples was ≥7.

50 ng of total RNA was amplified by applying Ovation RNA-Seq System V2 (NuGen, Emeryville, CA, USA) after which the resulting cDNAs were pooled in equal amount and the pool was used to prepare the DNA fragment library with SOLiD System chemistry (Life Technologies Corp., Carlsbad, CA, USA). Sequencing was performed using SOLiD 5500W platform and DNA sequencing chemistry (Life Technologies Corp., Carlsbad, CA, USA). Raw reads (75 bp) were color-space mapped to the human genome hg19 reference using Maxmapper algorithm implemented in the Lifescope software (Life Technologies, Ltd). Mapping to multiple locations was permitted. The quality threshold was set to 10, giving the mapping confidence was more than 90. Reads with score less than 10 were filtered out. Average mapping quality was 30. Analysis of the RNA content and gene-based annotation was done with whole-transcriptome workflow. Raw sequencing data with appropriate experimental information is available in the NCBI Gene Expression Omnibus (GEO) repository under the accession number GSE66511 (http://www.ncbi.nlm.nih.gov/geo/query/acc.cgi?acc=GSE66511).

### Statistical analysis

Patients and controls were tested for potential bias caused by covariates.

Non-normalized raw counts were used for the EdgeR package to perform differential gene expression analysis after quality control of samples. EdgeR performs model-based scale normalization, estimates dispersions and applies negative binomial model. EdgeR is very flexible tool for RNAseq data analysis to find differentially expressed genes [14,15]. It implements negative binomial model fitting followed by testing procedures for determining differential expression.

As our sample contains paired-samples (lesional and non-lesional skins are form the same persons) we used two different approaches. For the paired samples we applied general linear modelling where the subjects were added to the contrast matrix. GLM likelihood ratio test was applied for LP-NLP comparison. Different approach was applied for comparisons between LP-C and NLP-C. In this approach we used group-wise comparisons where negative binomial fitting was followed by exact test. False discovery rate (FDR) adjustment was used for multiple testing correction [16]. FDR threshold 0.1 for statistical significance was applied. Genes with larger differential expression were defined with logFC threshold 0.5 (i.e. 50% change between experimental conditions).

### Functional annotation of differentially expressed genes

The functional analysis of a gene network was used to identify the biological functions that are most significantly related to the molecules in the network. To define the functional networks of differentially expressed genes, data were analyzed by using the Ingenuity Pathway Analysis (IPA, Ingenuity Systems, *www.ingenuity.com*) that calculates a significance score (network score) for each network. This score indicates whether the likelihood that the assembly of a set of focus genes in a network could be explained by random chance alone (e.g., score of 2 indicates that there is a chance of 1 in 100 that the focus genes are together in a network due to random chance). A data set containing the gene identifiers and their corresponding fold change (log2) values were uploaded into the IPA software. Each gene identifier was mapped to its corresponding gene object in the Ingenuity Pathways Knowledge Base to identify molecules whose expression was significantly differentially regulated (focus genes or Networks Eligible molecules). These focus genes were overlaid onto a global molecular network developed from information contained in the Ingenuity Knowledge Base. Networks of these focus genes were then algorithmically generated based on their connectivity. A network represents the molecular relationships between genes or gene products, which are represented as nodes, and the biological relationship between two nodes is represented as an edge (line). All edges are supported by at least one reference from the literature, or from canonical information stored in the Ingenuity Pathways Knowledge Base. Fold change difference threshold for the functional analysis was set on 2 (log2 transformed).

### Quantitative real-time PCR

250 ng of total RNA was used with High Capacity RNA-to-cDNA Kit (Life Technologies Co, USA) for cDNA synthesis. QuantiTect Reverse Transcription Kit (Qiagen) was applied for cDNA synthesis in case of g1 or s1 assays. Both cDNA synthesis kits were used according to the manufacturer’s protocol. cDNA was used as a template for TaqMan qRT-PCR in 7900 Fast QRT-PCR System (Life Technologies Co). Two primers and labelled probe were used to detect the mRNA expression level of the reference gene hypoxanthine phosphoribosyl-transferase-1 (HPRT1; primer sequences available upon request). Expression levels of IL36G, IL36RN, LCE3D, IFI6, TGM1, SPRR2B, OAS2, CRABP2, S100A12 and PARP9 were detected with TaqMan Gene Expression Assays (Life Technologies Co) Hs00219742_m1, Hs01104220_g1, Hs00754375_s1, Hs00242571_m1, Hs01070310_m1, Hs01595682_s1, Hs00942643_m1, Hs00275636_m1, Hs00942835_g1 and Hs00967084_m1, respectively.

The relative gene expression levels from qRT-PCR were calculated relative to the reference gene HPRT-1 using 2^-ΔCT^ method. The normality of the data was tested with Shapiro test and based on its results unpaired *t*-test or Mann–Whitney test was used. We also performed correlation analysis to find the strength of expressional co-regulation of selected genes.

## Results

### The effect of covariates

Present study compared skin RNA expressional profiles in psoriasis patients with healthy controls (C). In addition, we compared the lesional (LP) and non-lesional (NLP) skin biopsies from psoriasis patients in order to find activity related genes. In order to exclude most common confounding variables, we tested for the differences in age, body weight, height, BMI and smoking status. There was no statistical difference between any of the studied covariate. In case of age (38 versus 41) the p-value was 0.61, for height (173 versus 174 cm) p-value was 0.77, for body weight (79 kg versus 87 kg) p-value was 0.28, for BMI (26.2 versus 29.0) p-value was 0.33. Also, the smoking status did not differ between the study groups (p-value 0.4).

### RNA sequencing results

**Figure 1:**
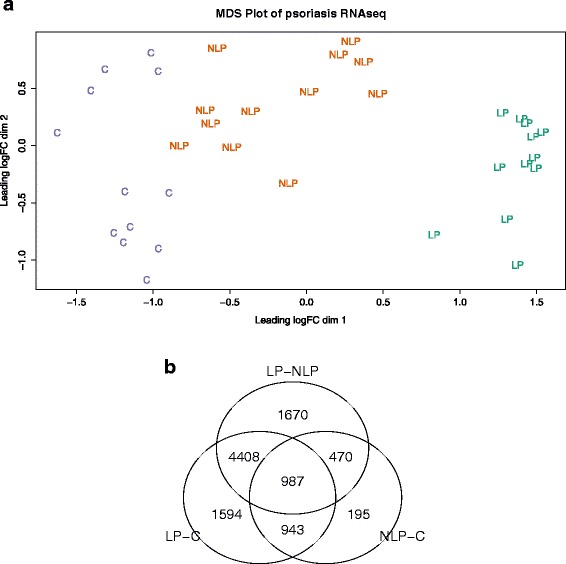
General illustration of study groups. **a**. Multidimensional scaling plot of the original data indicates clear separation of experimental groups – controls (C), non-lesional psoriatic skin (NLP) and lesional psoriatic skin (LP). This plot verifies good sampling and confirms reliability of collected data. **b**. Venn diagram for different comparisons between study groups.

RNA sequencing experiment gave high quality reads with good similarity between different samples (Figure 1a). Multidimensional scaling analysis indicated good separation of study groups based on the gene expression fold-change difference (Figure 1a). In Figure 1a the clear separation of LP and C samples is evident, whereas NLP samples are between LP and C. This is expected, as in the LP skin active inflammation is evident. NLP is a skin without inflammation, but nevertheless, non-lesional skin of psoriasis patients has molecular differences compared to the normal skin. Indeed, differential expression analysis found significant differences between all study groups.

We had three different groups in our model: lesional psoriatic skin (LP, n = 12) paired with the non-lesional psoriatic skin (NLP, n = 12) and normal skin from healthy controls (C, n = 12). We applied generalized linear model (GLM) for paired sample analysis. Comparison between other groups was done with the exact test (NLP versus C and LP versus C).

In the lesional psoriatic skin compared to the skin of healthy controls (LP-C), 7,932 genes were differentially expressed with the confidence level of FDR < 0.1 (for 30 genes Table 2, for all genes Additional file 1: Table S1). Applying of LogFC threshold verified that 5,853 genes are at least 50% different between LP-C (FDR < 0.1). 7,539 genes were differentially expressed (False Discovery Rate, FDR < 0.1) after pair-wise comparison of lesional psoriatic skin to the non-lesional skin of psoriasis patients (LP-NLP) (Additional file 2: Table S2). After logFC filtering 4,626 genes remained differentially expressed at least by 50% between LP-NLP. Comparison between non-lesional psoriatic skin and the skin of healthy controls (NLP-C) identified 2,595 genes to be differentially expressed with FDR < 0.1 (Additional file 3: Table S3). Additional logFC threshold indicated that 970 genes were 50% differentially expressed between NLP and C. The number of differentially expressed genes between different groups is illustrated in the Venn diagram (Figure 1b).

**Table 2:** **Differentially expressed genes in psoriatic lesions compared to healthy controls (LP-C)**

**Symbol**	**logFC**	**logCPM**	**PValue**	**FDR**	**Entrez Gene Name**
OAS2	4.43	7.16	9.26E-70	2.14E-65	2'-5'-oligoadenylate synthetase 2
PLA2G4E	2.55	7.60	2.74E-68	3.16E-64	Phospholipase A2, group IVE
KLK9	5.20	4.01	2.20E-67	1.69E-63	Kallikrein-related peptidase 9
S100A12	9.00	4.41	1.25E-65	7.20E-62	S100 calcium binding protein A12
LCE3E	6.99	4.45	6.02E-65	2.78E-61	Late cornified envelope 3E
TGM1	3.42	6.51	4.94E-61	1.90E-57	Transglutaminase 1
OAS3	2.99	7.25	1.01E-59	3.33E-56	2'-5'-oligoadenylate synthetase 3
PARP9	2.20	7.11	2.80E-59	8.08E-56	Poly (ADP-ribose) polymerase, 9
CRABP2	2.49	5.65	1.99E-57	5.10E-54	Cellular retinoic acid binding protein 2
PLA2G4D	4.33	6.10	3.03E-56	7.01E-53	Phospholipase A2, group IVD
IL1F9	5.64	6.61	2.26E-55	4.74E-52	Interleukin 36, gamma
ALOX12B	3.01	7.30	7.12E-55	1.37E-51	Arachidonate 12-lipoxygenase
SAMD9	3.01	6.23	8.59E-55	1.53E-51	Sterile alpha motif domain
IL1F5	3.59	7.91	1.14E-53	1.87E-50	Interleukin 36 receptor antagonist
C10orf99	6.22	5.44	3.20E-52	4.93E-49	Chrom 10 open reading frame 99
DEFB4A	10.43	6.59	6.30E-52	9.09E-49	Defensin. beta 4A
AKR1B10	6.22	4.39	4.02E-51	5.46E-48	Aldo-keto reductase family 1
PAPL	4.01	4.60	1.56E-50	2.01E-47	Iron/zinc purple acid protein
GLTP	2.01	7.42	5.00E-50	6.08E-47	Glycolipid transfer protein
KDM6B	−1.36	11.51	1.48E-49	1.71E-46	Lysine (K)-specific demethylase 6B
KYNU	4.49	4.72	2.54E-48	2.79E-45	Kynureninase
RRM2	2.68	5.47	3.29E-48	3.46E-45	Ribonucleotide reductase M2
ZC3H12A	3.06	4.88	2.30E-47	2.31E-44	Zinc finger CCCH-type cont 12A
SDR9C7	2.70	5.00	6.01E-47	5.79E-44	Short chain dehydrogenase/reductase
HPSE	3.47	4.17	1.31E-46	1.21E-43	Heparanase
APOL6	2.23	6.52	1.91E-46	1.69E-43	Apolipoprotein L, 6
NIPAL4	1.39	9.13	6.90E-46	5.90E-43	NIPA-like domain containing 4
DMD	−1.81	7.46	9.69E-46	7.99E-43	Dystrophin
S100A7A	10.02	10.30	2.84E-45	2.26E-42	S100 calcium binding protein A7A

LogFC is fold changes differences in log_2_ scale and it describes how many times gene expression differs between groups. Positive values indicate up-regulation in psoriasis. LogCPM (log_2_ counts-per-million) is average gene expression signal in all samples. FDR is genome-wide corrected P-value.

**Figure 2:**
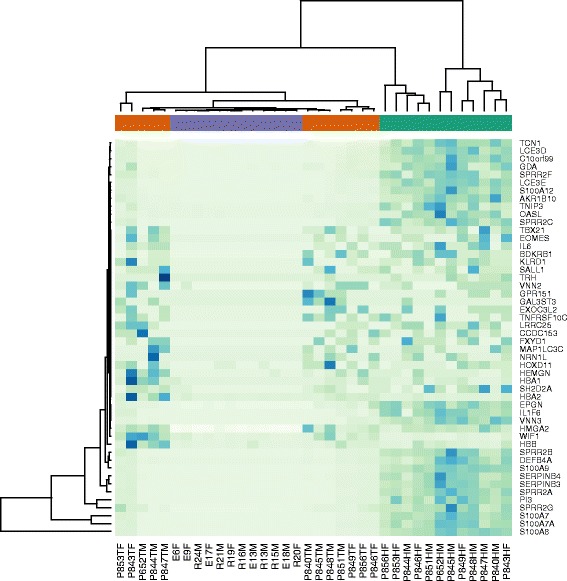
Heatmap of the 50 genes with largest fold change differences between NLP and C samples. Violet bar is for control samples, red bar is for non-lesional samples and green bar is for lesional skin samples.

To illustrate the observed differences and their relations to the skin conditions, we performed cluster analysis of the entire sample. Heatmap (Additional file 4: Figure S2) of top 200 differing genes between LP and C samples with the lowest FDR values were generated. Moreover, we generated heatmaps from smaller sample subset based on their gene expression fold-changes (Figure 2, Additional file 5: Figure S1). Again, our samples were distinguishable based on the gene expression data what confirms the quality of RNAseq data. An interesting pattern was found in Figure 2. This is comparison of NLP-C and therefore should illustrate the genes that are different in non-lesional and control sample. NLP sample has genes that have similar expressional pattern to the lesional sample (central block of genes in Figure 2) and about the same amount of genes that have similar expressional pattern to the control sample (two blocks in the upper and lower part of Figure 2). The genes with expressional pattern similar to the lesional sample, represent the genes that form the molecular signature of psoriasis in non-lesional skin. These genes can be described as “dormant pathology” or “background inflammation”, indicating certain standby situation in non-lesional skin. Interestingly, among these genes are IL1F6 and IL6. IL6 is well known to be involved in the pathogenesis of psoriasis [17,18]. IL1F6 (IL36A) is a gene that was recently found to be part of the signalling system what is active in psoriasis [19,20].

**Figure 3:**
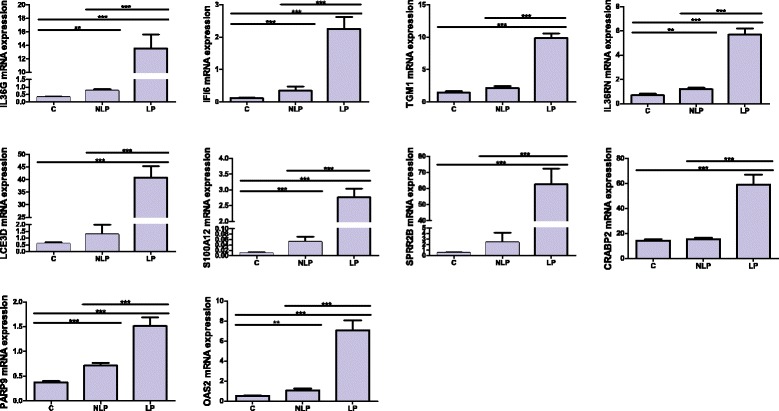
Quantitative real-time PCR analysis confirmed transcriptional differences found with RNA-seq. In case of TGM1, LCE3D and SPRR2B there is no difference between C and NLP. *** - p < 0.001. ** - p < 0.01.

### Functional analysis of gene expression networks

Ingenuity pathway analysis software was used for functional analysis. We analysed the genes differentially expressed between all three groups and performed three separate analyses. This enabled us to describe the functional networks that characterize non-lesional skin and lesional skin. Comparison between lesional skin (LP) and healthy controls (C) revealed activation of the “Granulocyte adhesion and diapedesis” (Table 3) as the top enriched canonical pathway (p = 2.81E-20). In addition, “Agranylocyte adhesion and diapedesis” (p = 6.08E-13) and “Role of IL-17A in Psoriasis” (p = 8.52E-11) were two other highly enriched canonical pathways in LP compared to C sample. In addition to the IL-17 signalling, canonical pathway “IL-10 signalling” was also significantly enriched in the lesional samples compared to control samples. Activation of IL-10 signalling means that IL19, IL20, IL24 and IL26 were significantly up regulated, whereas IL-10 was not. Activation of IL10 canonical signalling was not found in NLP samples.

**Table 3:** **Activated canonical pathways in LP-C comparisons**

**Ingenuity canonical pathways**	**-log(p-value)**	**Ratio**
Granulocyte Adhesion and Diapedesis	19.60	0.31
Agranulocyte Adhesion and Diapedesis	12.20	0.25
Role of IL-17A in Psoriasis	10.10	0.79
Atherosclerosis Signaling	9.75	0.25
Pathogenesis of Multiple Sclerosis	9.73	0.90
Differential Regulation of Cytokine Production in Intestinal Epithelial Cells by IL-17A and IL-17F	9.54	0.61
Differential Regulation of Cytokine Production in Macrophages and T Helper Cells by IL-17A and IL-17F	8.91	0.67
Role of Hypercytokinemia/hyperchemokinemia in the Pathogenesis of Influenza	8.38	0.39
LXR/RXR Activation	6.29	0.20
Role of Pattern Recognition Receptors in Recognition of Bacteria and Viruses	6.13	0.22
Role of Cytokines in Mediating Communication between Immune Cells	5.89	0.31
Altered T Cell and B Cell Signaling in Rheumatoid Arthritis	5.85	0.22
T Helper Cell Differentiation	5.66	0.26
Hepatic Fibrosis / Hepatic Stellate Cell Activation	5.03	0.18
Communication between Innate and Adaptive Immune Cells	4.86	0.19
IL-10 Signaling	3.95	0.21

Differentially expressed genes were used as a “signature” to find what biological function is changed in the skin of psoriasis patients.

Comparison between LP and NLP samples (LP-NLP) indicated the enrichment of the “Granulocyte adhesion and diapedesis” as the top canonical pathway (p = 1.13E-13, Additional file 6: Table S4), similarly to the LP-C analysis. LP samples also exhibited significant activation of the canonical pathway “Role of cytokines in mediating communication between immune cells” (p = 2.04E-11). Comparison between NLP and C samples found up-regulation of canonical pathway “Role of IL-17A in Psoriasis” (p = 2.31E-08) (Additional file 7: Table S5). However, the enrichment was not that large as in case of lesional skin and the number of differentially expressed genes was reduced on NLP-C comparison (Additional file 3: Table S3). Therefore, differences were more quantitative than qualitative (Additional file 8: Figure S4 and Additional file 9: Figure S3. However, there were some gene sets forming quite distinctive pattern or footprint characteristic only for the LP sample (Additional file 9: Figure S3). For instance, the genes LCE3E, SPRR2B, DEFB4A, S100A7A, S100A8, SERPINB4 and S100A12 were specific only for the lesional and not found in non-lesional sample (Figure 3). Activation of “IL-10 signalling” was also found only in the LP samples. On the other hand, IL1F6, IL6, GPR151, WIF1 and TNFRSF10C were activated in both lesional and non-lesional samples (Figure 3). Therefore, there are genes characteristic for the lesional skin and there are genes what are activated also in the skin without psoriatic lesion. The functional impact of non-lesional transcriptional signature needs further studies.

### Confirmative quantitative real-time PCR

In order to verify the RNA seq findings we performed quantitative real-time PCR analysis for 10 genes. We analysed the expression of IL1F5 (IL36RN) and IL1F9 (IL36G) genes and found that their expression profile correlated very well with the inflammation in the skin. The results from qRT-PCR were similar with RNA-seq data and the differences in gene expression levels followed completely the skin status – C, NLP or LP. Only in case of TGM1, LCE3D and SPRR2B genes there wasn’t statistically significant difference between NLP and C, but still the difference between LP-C and LP-NLP was significant (Figures 3). These results support that LCE3D and SPRR2B are genes more characteristic for the psoriatic lesion, than for the non-lesional skin. Correlation analysis indicated very high and significant correlation between the analysed genes (Additional file 10: Figure S6).

## Discussion

In present study we performed whole transcriptome analysis of lesional and non-lesional psoriasis skin samples and compared them with the healthy control samples. There are several previous studies where transcriptome changes in psoriasis patients have been analysed [8-11,21-27]. In most of these studies hybridization based genechips were used. However, recently several additional papers reported the results of RNA-seq based transcriptome analysis [9,12,13,28,29]. One of these studies was focused on the differences in the small RNAs [28]. The other studies analysed whole genome transcriptome [9,12,13,29]. One major difference between RNA-seq and genechip based transcriptome studies is the substantially larger number of differentially expressed genes and the appearance of the genes previously not discovered in similar studies. This difference can be explained by the lower detection limit and wider dynamic range that is characteristic for RNA-seq. However, the majority of changes described in the genechip based studies and in published RNA-seq studies are quite similar. Most of the findings described in our paper coincide with previously published large scale RNA-seq study [12]. However, we applied slightly different approach and we also found some interesting targets not described in previous studies. We used total RNA (not only polyA RNA), we applied only gene-level analysis and functional annotation of the networks related to the differential transcriptome signatures. Also, the statistical approaches in previous studies have been slightly different. However, despite these technical differences, the results of our study and previous studies are generally similar.

Our study revealed significant and substantial differences between all three groups in their transcriptome profile. Most remarkably, oligoadenylate synthetases (OAS2 and OAS3) and phospholipases from A2 (PLA2G4E and PLA2G4D) group were found up regulated in lesional psoriatic skin. OAS2 and OAS3 are related to the innate immunity and antiviral response [30]. Recent studies have shown the association of OAS genes with psoriasis and systemic lupus erythematosus [31,32]. OAS genes have thoroughly been discussed as part of the IFN signature in several studies focusing on psoriasis [24,33]. Phospholipases A2 are enzymes related to the metabolism of fatty acids in membrane phospholipids and related to the inflammatory signalling pathways [34]. Lipid metabolism and antimicrobial defence pathways are shown activated during psoriasis [10]. Moreover, OAS and PLA genes have also been found in other psoriasis transcriptome studies [12,29]. Therefore, these genes are clearly linked with the psoriasis and their general role in the chronic inflammatory conditions is shown in previous papers.

Another interesting set of genes we found activated during psoriasis are interleukin-36 (IL36) cytokines that belong to interleukin-1 family (IL1F). Namely, with very high confidence (FDR below 1E-50) we found IL1F9 (IL36G) and IL1F5 (IL36RN) up regulated in lesional skin samples. Quantitative real-time PCR verified our findings and even confirmed IL36G and IL36RN expression to be significantly associated with the skin condition. Expression was statistically different even between C and NLP skin samples, in LP samples the expression was even more elevated. IL36G is a recently discovered novel member of IL-1 family of cytokines [35]. IL36G has been shown to be involved in innate immunity and all IL36 cytokines (except IL36RN) have proinflammatory activity [36-38]. We can consider the activation of IL36G as the most significant finding and propose the family of these genes to be important biomarkers for psoriasis. Indeed, one recent study identified IL36G as the most outstanding biomarker for psoriasis [39]. Several other studies confirm the involvement of IL36 cytokines in the psoriasis [19,36,40]. More precisely, IL36G was recently identified as one of the 13 hallmark psoriasis genes universally or near-universally up-regulated in psoriasis lesions [40]. IL-1 family members play a key role in the function of innate and adaptive immunity and are new promising targets for immunpathologies [41]. In our study we only found IL36 cytokines (IL36G, IL36RN and IL36A) to be related to psoriasis. IL37 (IL1F7) was up-regulated only in the non-lesional psoriatic skin, which makes its role in psoriasis very intriguing. In another previous study using RNA-seq for psoriasis samples the IL37 was found to be down-regulated in lesional skin [12]. Therefore, our results confirm previous studies, that IL36 genes have significant role in regulation of psoriasis and psoriasis activity.

Pathway analysis found clear activation of innate immune response and inflammation. Pathway “Role of IL-17A in Psoriasis” was enriched in both LP-C and NLP-C comparisons (Table 3 and Table 4). The finding that the IL-17 pathway is activated during psoriasis is not new and suits with findings from previous studies [42]. Moreover, the IL36 cytokine discussed in previous section is also linked to the IL-17 network. Granulocyte and agranulocyte adhesion canonical pathways illustrate the activation of inflammatory pathways and inflammatory conditions. Additional file 8: Figure S4 illustrates gene expression profiles compared between LP and C samples. It is visible that the majority of genes are up-regulated and they are involved in inflammation. Comparison of Additional file 8: Figures S4, Additional file 9: Figure S3 and Additional file 11: Figure S5 illustrates how the number of activated genes is decreasing as inflammatory conditions getting milder (from lesional to non-lesional and healthy skin). Additional file 9: Figure S3 has remarkably less activated genes from the “Psoriasis” genetic network. On the other hand, the Additional file 10: Figure S6 illustrates genes that are related to the background inflammation in the non-lesional psoriatic skin. Most well known psoriasis related genes activated in non-lesional skin are IL6, IL22, IL36A, IL36G, IL19, IL20, S100A7 and S100A12. One previous study has also described similar “pre-psoriatic” gene expression signature [10]. In accordance with this study, we found that the gene expression differences between LP and C samples were more prominent than differences between NLP and C samples. We can conclude that non-lesional skin in psoriasis patients, while considered normal, still contains molecular signature characteristic for psoriasis.

**Table 4:** **Activated canonical pathways in NLP-C comparisons**

**Ingenuity canonical pathways**	**-log(p-value)**	**Ratio**
Role of IL-17A in Psoriasis	7.64	0.50
γ-linolenate Biosynthesis II (Animals)	4.05	0.21
Granulocyte Adhesion and Diapedesis	3.80	0.08
Role of Hypercytokinemia/hyperchemokinemia in the Pathogenesis of Influenza	3.62	0.15
Atherosclerosis Signaling	3.10	0.08
Altered T Cell and B Cell Signaling in Rheumatoid Arthritis	3.09	0.09
Agranulocyte Adhesion and Diapedesis	3.02	0.07
Role of Cytokines in Mediating Communication between Immune Cells	3.02	0.13
Differential Regulation of Cytokine Production in Macrophages and T Helper Cells by IL-17A and IL-17F	2.82	0.22
LPS/IL-1 Mediated Inhibition of RXR Function	2.72	0.06
Stearate Biosynthesis I (Animals)	2.51	0.10
Primary Immunodeficiency Signaling	2.44	0.09
Differential Regulation of Cytokine Production in Intestinal Epithelial Cells by IL-17A and IL-17F	2.41	0.17
IL-17 Signaling	2.34	0.09
Communication between Innate and Adaptive Immune Cells	2.33	0.07
Uracil Degradation II (Reductive)	2.32	0.18

Differentially expressed genes were used as a “signature” to find what biological function is changed in the skin of psoriasis patients.

Difference between NLP and C samples may be caused by the confounding covariates that should be considered. After analysis of the distribution of covariates in study groups we found no statistical difference in age (p = 0.61), body weight (p = 0.30), height (p = 0.77), BMI (p = 0.32) and smoking status (p = 0.41) between controls and patients. Based on these data we can assume, that the influence of covariates is minimal and the transcriptional differences are caused by the psoriasis.

Taken together, the most of the genes we found have also been identified in earlier studies. Here we described results of complex analysis of the transcriptional signatures characteristic for lesional and non-lesional psoriatic skin and normal skin. There are several published results on the transcriptional changes of the psoriasis and in large extend the results are coinciding. However, differences exist in technical details (total RNA versus polyA RNA), statistical models and study designs (lesional versus non-lesional or lesional-non-lesional-normal). Our study design allowed us to use wider model (comparison between three conditions) and to gain more detailed information about the psoriasis. We found significant activation of IL36 cytokines, what has not been described in previous studies. Moreover, we found that IL17 related cytokines are also activated in non-lesional psoriatic biopsies indicating more general immune pathology in the skin. Therefore, even the skin looks noormal, immune system inside the skin is still altered.

## Conclusions

We found the genes of IL36 cytokine family to be involved in the pathogenesis of psoriasis. These genes have received very little attention in previous research and therefore, further exploration of their regulation can give new insights to the psoriasis.

### Availability of supporting data

The data set supporting the results of this article is available in the NCBI Gene Expression Omnibus (NCBI-GEO) repository. The accession number for dataset is GSE66511 (http://www.ncbi.nlm.nih.gov/geo/query/acc.cgi?acc=GSE66511).

## Additional files

Additional file 1: Table S1. The list of 500 most significant differentially expressed genes based on comparison of LP to the C. LogFC is fold changes differences in log_2_ scale and it describes how many times gene expression differs between groups. Positive values indicate up-regulation in psoriasis. LogCPM (log_2_ counts-per-million) is average gene expression signal in all samples. FDR is genome-wide corrected P-value. Additional file 2: Table S2. The list of 500 most significant differentially expressed genes based on comparison of LP to the NLP skin. LogFC is fold changes differences in log_2_ scale and it describes how many times gene expression differs between groups. Positive values indicate up-regulation in psoriasis. LogCPM (log_2_ counts-per-million) is average gene expression signal in all samples. FDR is genome-wide corrected P-value. Additional file 3: Table S3. The list of 500 most significant differentially expressed genes based on comparison of NLP skin to the C skin. LogFC is fold changes differences in log_2_ scale and it describes how many times gene expression differs between groups. Positive values indicate up-regulation in psoriasis. LogCPM (log_2_ counts-per-million) is average gene expression signal in all samples. FDR is genome-wide corrected P-value. Additional file 4: Figure S2. Heatmap of the 50 genes with largest fold change differences after pairwise comparison of LP and NLP samples. Violet bar is for control samples, red bar is for non-lesional samples and green bar is for lesional skin samples. Additional file 5: Figure S1. Heatmap of the 50 genes with largest fold change differences between LP and C samples. Violet bar is for control samples, red bar is for non-lesional samples and green bar is for lesional skin samples. Additional file 6: Table S4. Activated canonical pathways in LP sample compared to NLP sample. Additional file 7: Table S5. Activated canonical pathways in NLP-C comparions. Additional file 8: Figure S4. Activated gene expression network related to psoriasis based on the differential gene expression profile between LP and NLP. The gene expression network was constructed based on their biological role. The figure illustrates expressional changes (red genes are higher, green genes are lower expressed) of particular genes and their cellular location (extracellular, membrane, cytoplasm or nucleus) in relation with other genes in particular network. Additional file 9: Figure S3. Activated psoriasis-related gene expression network based on the differential gene expression profile between LP and C. The gene expression network was constructed based on their biological role. The figure illustrates expressional changes (red genes are higher, green genes are lower expressed) of particular genes and their cellular location (extracellular, membrane, cytoplasm or nucleus) in relation with other genes in particular network. Additional file 10: Figure S6. Correlation analysis of the genes analysed with quantitative real-time PCR. Additional file 11: Figure S5. Activated psoriasis gene expression network based on the differential gene expression profile between NLP and C. The gene expression network was constructed based on their biological role. The figure illustrates expressional changes (red genes are higher, green genes are lower expressed) of particular genes and their cellular location (extracellular, membrane, cytoplasm or nucleus) in relation with other genes in particular network.

## Abbreviations

LP: Lesional psoriatic skin
NLP: non-lesional psoriatic skin
C: normal skin of healthy controls
