# Supplementary figures and images for: Transcriptional landscape of psoriasis identifies the involvement of IL36 and IL36RN
Source: BMC Genomics. 2015 Apr 19;16(1):322. doi: 10.1186/s12864-015-1508-2 (PMC4405864; doi:10.1186/s12864-015-1508-2)

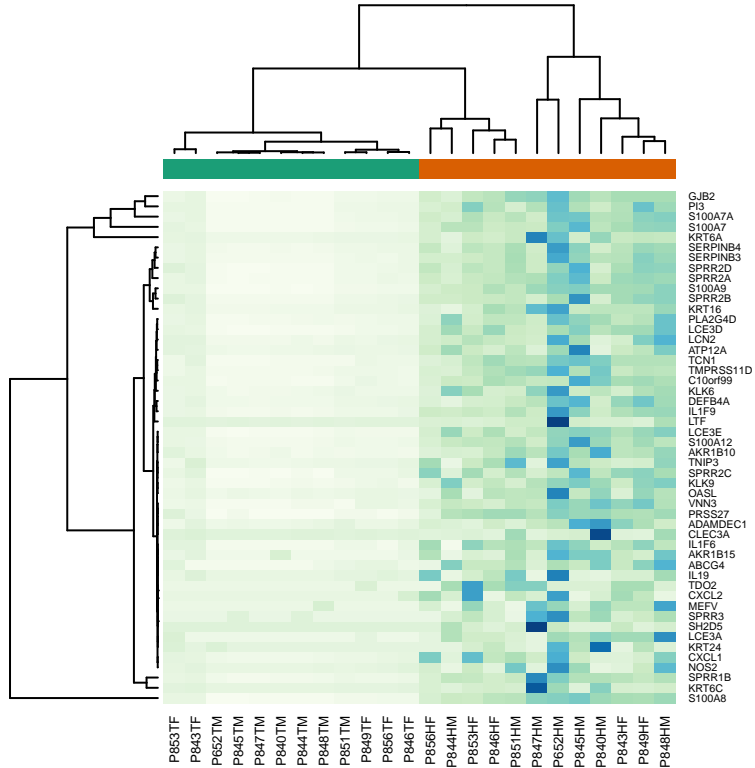

Supplement: Additional file 4: Figure S2. — Heatmap of the 50 genes with largest fold change differences after pairwise comparison of LP and NLP samples. Violet bar is for control samples, red bar is for non-lesional samples and green bar is for lesional skin samples. [file 12864_2015_1508_MOESM4_ESM.pdf]

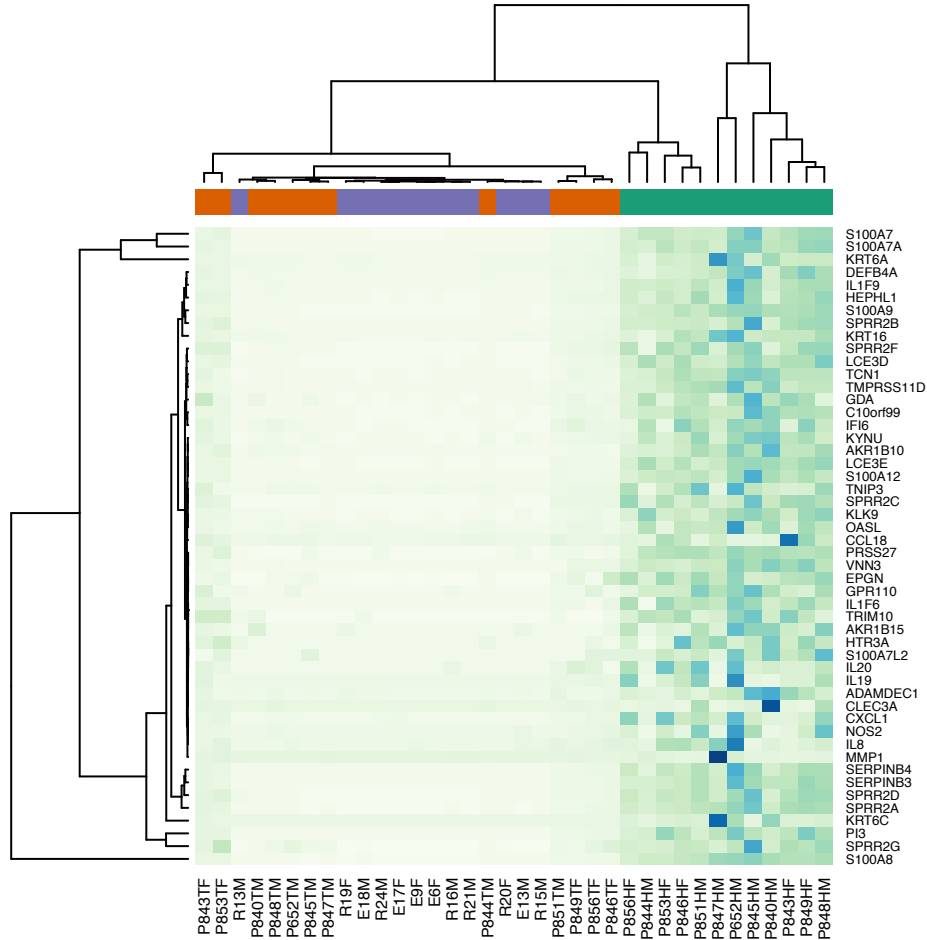

Supplement: Additional file 5: Figure S1. — Heatmap of the 50 genes with largest fold change differences between LP and C samples. Violet bar is for control samples, red bar is for non-lesional samples and green bar is for lesional skin samples. [file 12864_2015_1508_MOESM5_ESM.pdf]

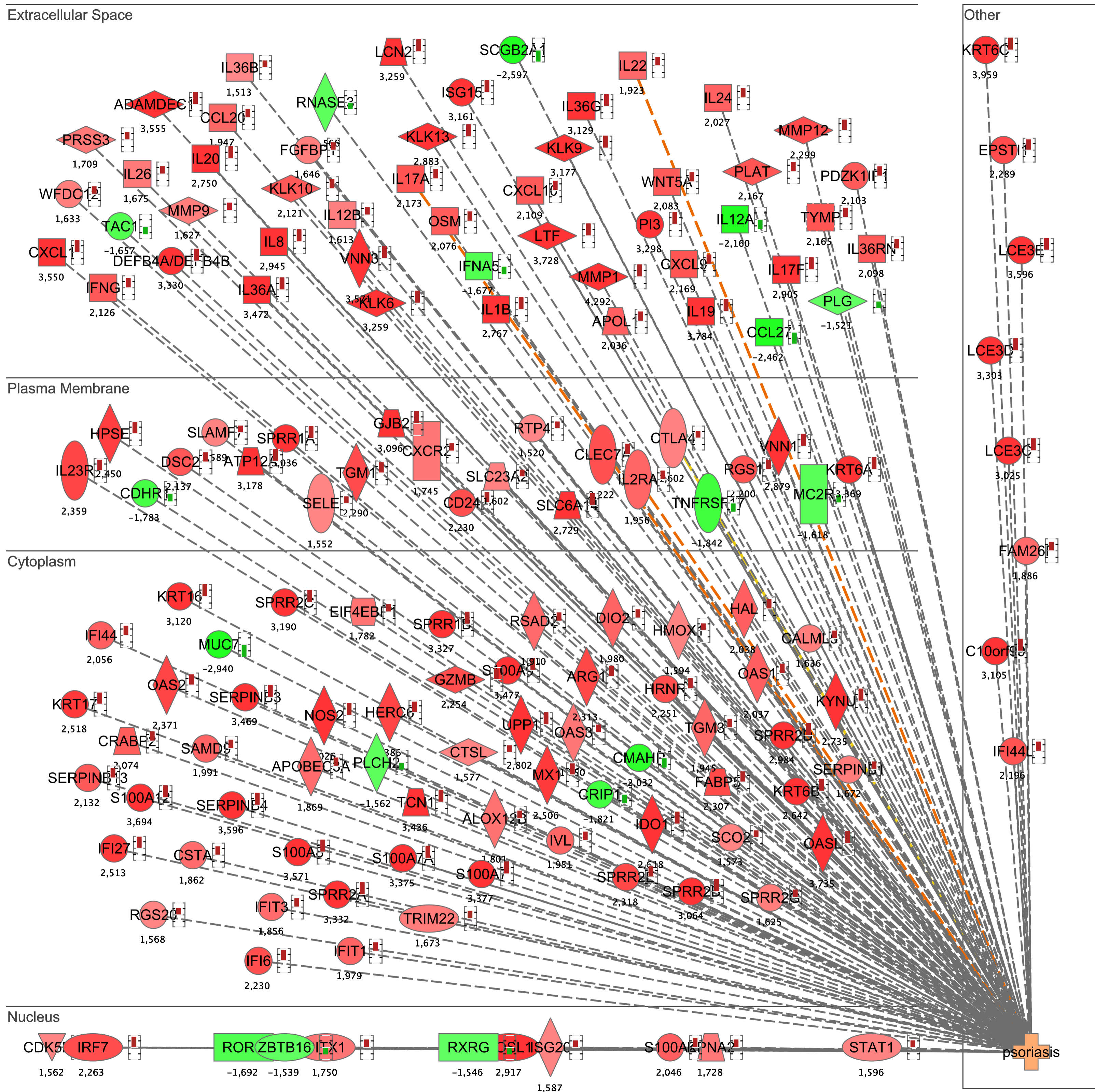

Supplement: Additional file 8: Figure S4. — Activated gene expression network related to psoriasis based on the differential gene expression profile between LP and NLP. The gene expression network was constructed based on their biological role. The figure illustrates expressional changes (red genes are higher, green genes are lower expressed) of particular genes and their cellular location (extracellular, membrane, cytoplasm or nucleus) in relation with other genes in particular network. [file 12864_2015_1508_MOESM8_ESM.pdf]

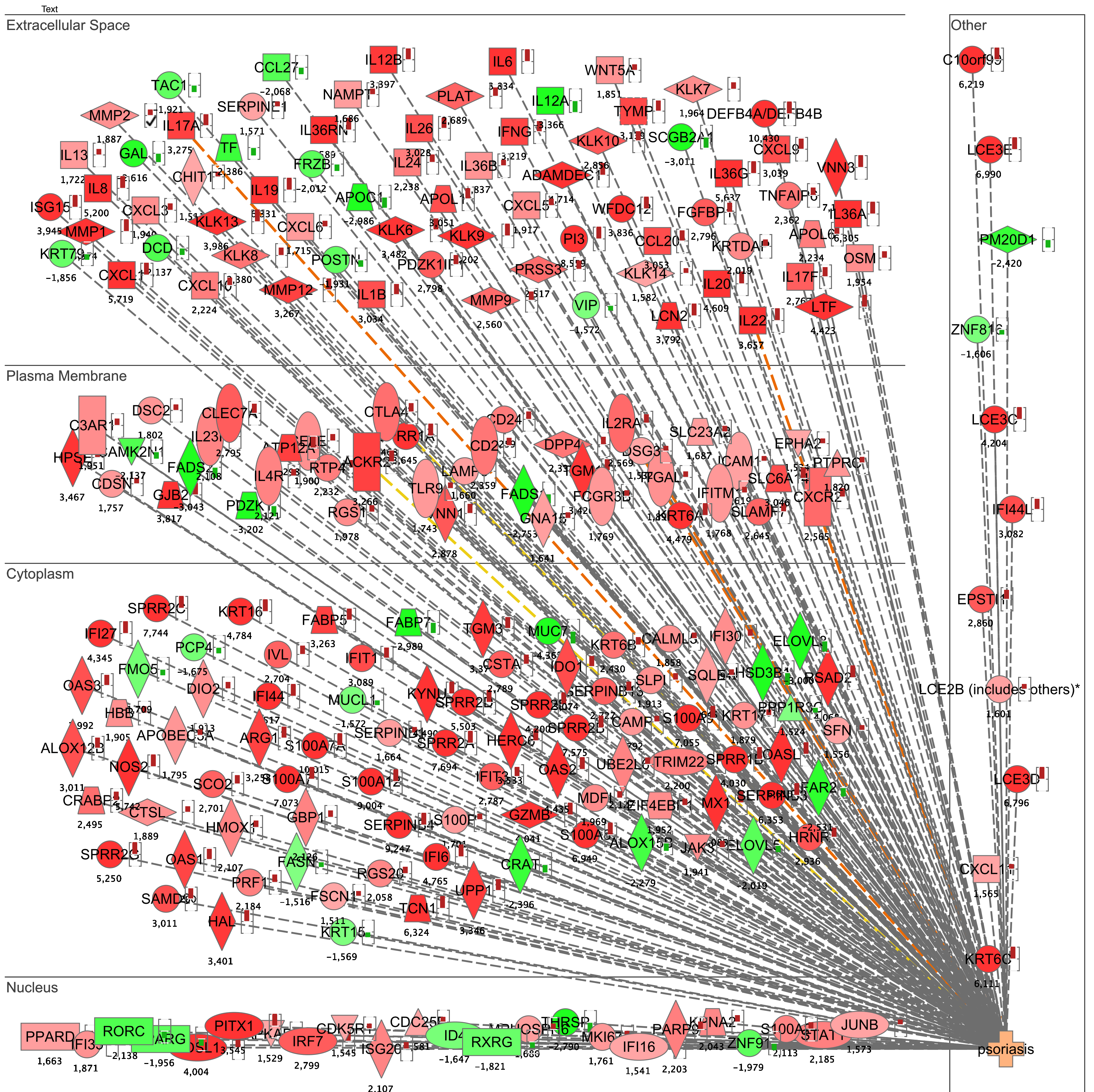

Supplement: Additional file 9: Figure S3. — Activated psoriasis-related gene expression network based on the differential gene expression profile between LP and C. The gene expression network was constructed based on their biological role. The figure illustrates expressional changes (red genes are higher, green genes are lower expressed) of particular genes and their cellular location (extracellular, membrane, cytoplasm or nucleus) in relation with other genes in particular network. [file 12864_2015_1508_MOESM9_ESM.pdf]

Spearman  $r=0,8322$   
 $p=0,0008$

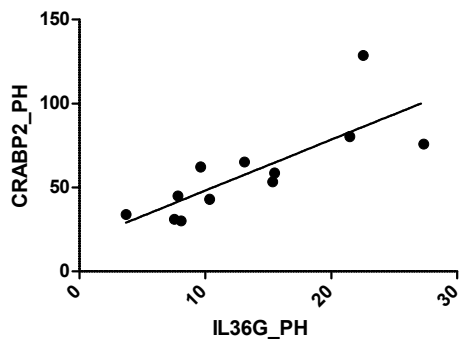

Pearson  $r=0,8186$   
 $p=0,0011$

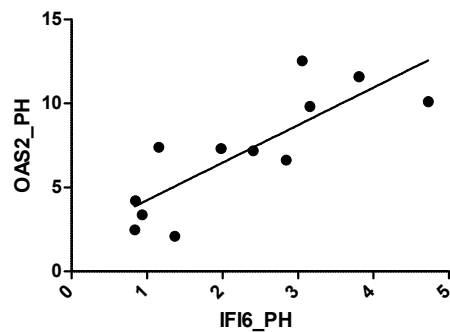

Pearson  $r=0,8976$   
 $p<0,0001$

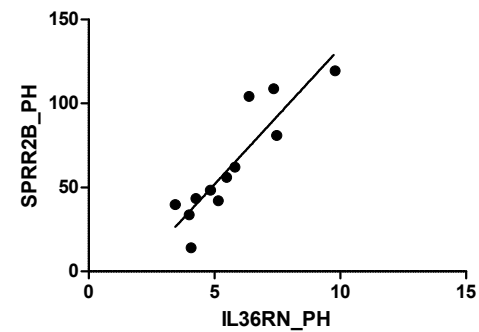

Pearson  $r=0,8718$   
 $p=0,0002$

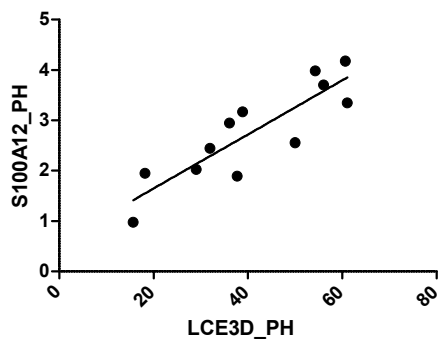

Spearman  $r=0,8741$   
 $p=0,0002$

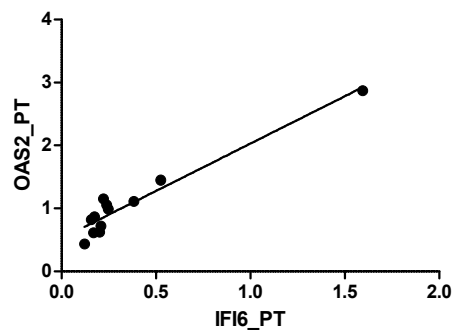

Pearson  $r=0,8764$   
 $p=0,0002$

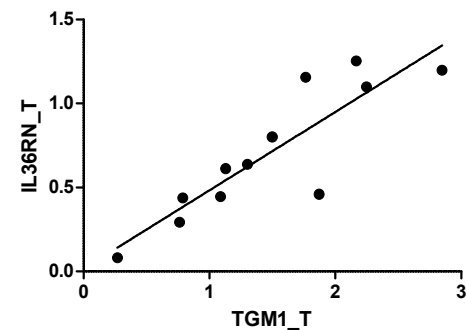

Pearson  $r=0,8467$   
 $p=0,0005$

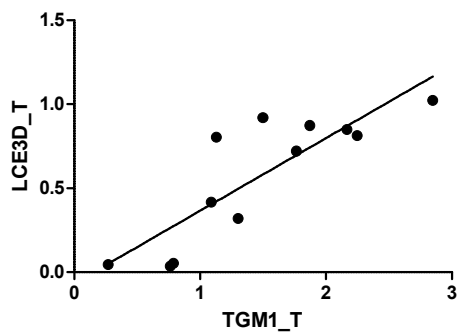

Pearson  $r=0,8874$   
 $p=0,0001$

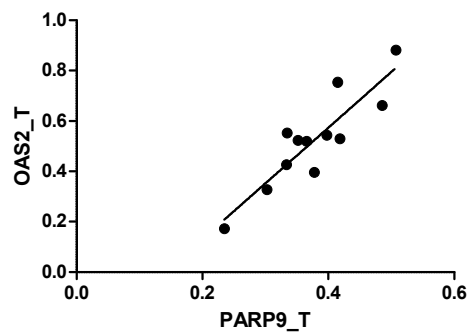

Supplement: Additional file 10: Figure S6. — Correlation analysis of the genes analysed with quantitative real-time PCR. [file 12864_2015_1508_MOESM10_ESM.pdf]

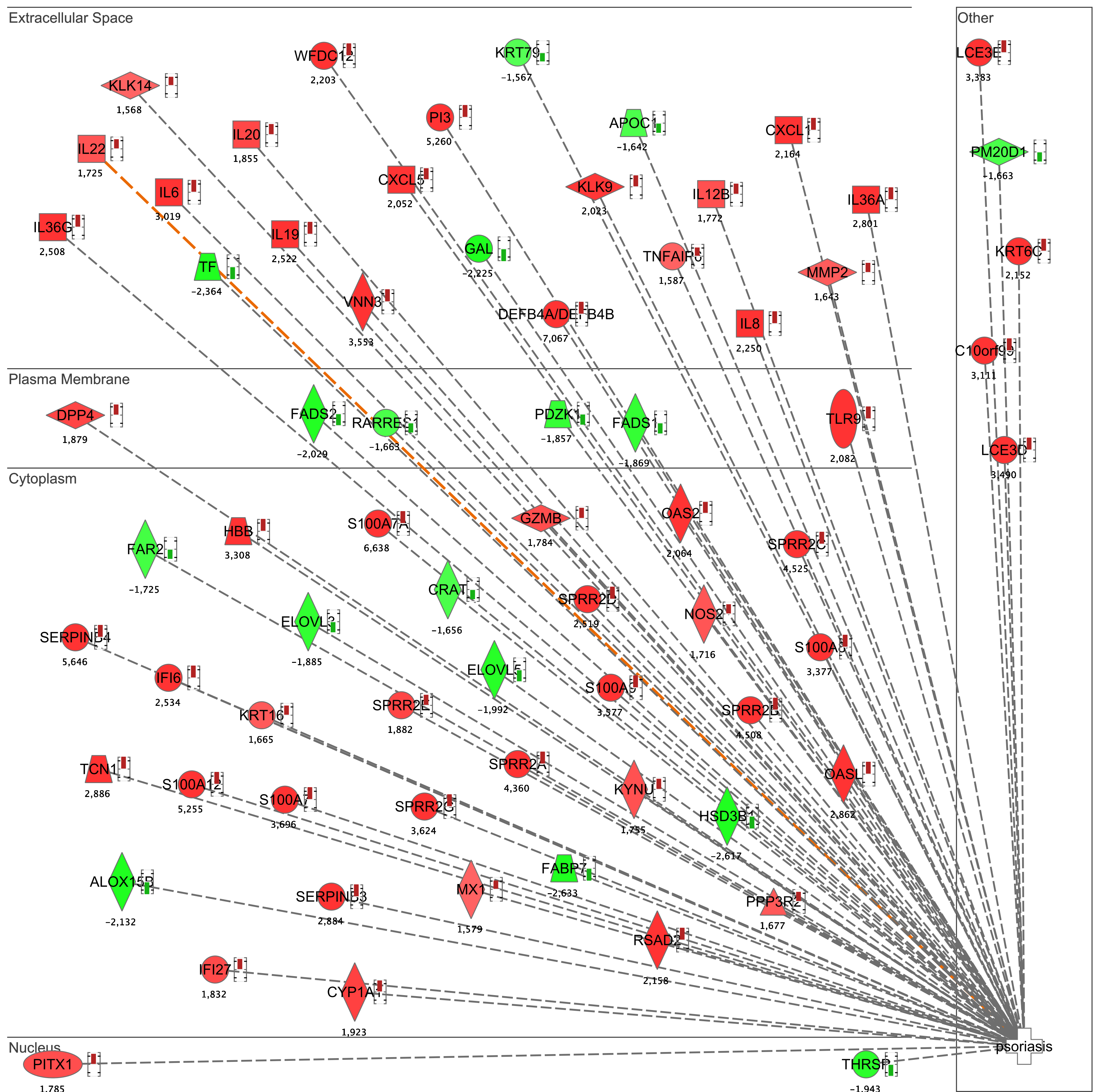

Supplement: Additional file 11: Figure S5. — Activated psoriasis gene expression network based on the differential gene expression profile between NLP and C. The gene expression network was constructed based on their biological role. The figure illustrates expressional changes (red genes are higher, green genes are lower expressed) of particular genes and their cellular location (extracellular, membrane, cytoplasm or nucleus) in relation with other genes in particular network. [file 12864_2015_1508_MOESM11_ESM.pdf]
