# Supplementary material for: Transcriptional landscape of psoriasis identifies the involvement of IL36 and IL36RN
Source: BMC Genomics. 2015 Apr 19;16(1):322. doi: 10.1186/s12864-015-1508-2 (PMC4405864; doi:10.1186/s12864-015-1508-2)
Supplement: Additional file 6: Table S4. — Activated canonical pathways in LP sample compared to NLP sample. [file 12864_2015_1508_MOESM6_ESM.pdf]

Table 4S. Activated canonical pathways in LP-NLP comparions

Ingenuity Canonical Pathways	-log(p-value)	Ratio
Granulocyte Adhesion and Diapedesis	12,90	0,19
Role of Cytokines in Mediating Communication between Immune Cells	10,70	0,33
Differential Regulation of Cytokine Production in Intestinal Epithelial Cells by IL-17A and IL-17F	10,20	0,52
Role of Hypercytokinemia/hyperchemokinemia in the Pathogenesis of Influenza	9,30	0,33
Agranulocyte Adhesion and Diapedesis	8,79	0,16
Role of IL-17A in Psoriasis	7,69	0,57
Differential Regulation of Cytokine Production in Macrophages and T Helper Cells by IL-17A and IL-17F	7,53	0,50
Atherosclerosis Signaling	7,46	0,16
LXR/RXR Activation	6,86	0,15
Communication between Innate and Adaptive Immune Cells	6,85	0,16
Altered T Cell and B Cell Signaling in Rheumatoid Arthritis	5,79	0,16
Role of Pattern Recognition Receptors in Recognition of Bacteria and Viruses	4,51	0,14
Role of Macrophages, Fibroblasts and Endothelial Cells in Rheumatoid Arthritis	4,50	0,09
T Helper Cell Differentiation	4,19	0,17
Graft-versus-Host Disease Signaling	3,68	0,18
Role of Osteoblasts, Osteoclasts and Chondrocytes in Rheumatoid Arthritis	3,67	0,09
