# Supplementary material for: Transcriptional landscape of psoriasis identifies the involvement of IL36 and IL36RN
Source: BMC Genomics. 2015 Apr 19;16(1):322. doi: 10.1186/s12864-015-1508-2 (PMC4405864; doi:10.1186/s12864-015-1508-2)
Supplement: Additional file 7: Table S5. — Activated canonical pathways in NLP-C comparions. [file 12864_2015_1508_MOESM7_ESM.pdf]

Table 5S. Activated canonical pathways in NLP-C comparions

| Ingenuity Canonical Pathways | -log(p-value) | Ratio |
|---|---|---|
| Role of IL-17A in Psoriasis | 7,64 | 0,50 |
| γ-linolenate Biosynthesis II (Animals) | 4,05 | 0,21 |
| Granulocyte Adhesion and Diapedesis | 3,80 | 0,08 |
| Role of Hypercytokinemia/hyperchemokinemia in the Pathogenesis of Influenza | 3,62 | 0,15 |
| Atherosclerosis Signaling | 3,10 | 0,08 |
| Altered T Cell and B Cell Signaling in Rheumatoid Arthritis | 3,09 | 0,09 |
| Agranulocyte Adhesion and Diapedesis | 3,02 | 0,07 |
| Role of Cytokines in Mediating Communication between Immune Cells | 3,02 | 0,13 |
| Differential Regulation of Cytokine Production in Macrophages and T Helper Cells by IL-17A and IL-17F | 2,82 | 0,22 |
| LPS/IL-1 Mediated Inhibition of RXR Function | 2,72 | 0,06 |
| Stearate Biosynthesis I (Animals) | 2,51 | 0,10 |
| Primary Immunodeficiency Signaling | 2,44 | 0,09 |
| Differential Regulation of Cytokine Production in Intestinal Epithelial Cells by IL-17A and IL-17F | 2,41 | 0,17 |
| IL-17 Signaling | 2,34 | 0,09 |
| Communication between Innate and Adaptive Immune Cells | 2,33 | 0,07 |
| Uracil Degradation II (Reductive) | 2,32 | 0,18 |
